# Manual of Materia Medica and Therapeutics: Being an Abridgement of the Late Dr. Pereira’s Elements of Materia Medica, Arranged in Conformity with the British Pharmacopœia

**Published:** 1866-10

**Authors:** 


MANUAL OF MATERIA MEDICA AND THERAPEUTICS: BEING AN ABRIDGEMENT OF THE LATE DR. PEREIRA'S ELEMENTS OF MATERIA MEDICA, ARRANGED IN CONFORMITY WITH THE BRITISH PHARMACOPŒIA, AND ADAPTED TO THE USE OF MEDICAL PRACTITIONERS, CHEMISTS AND DRUGGISTS, MEDICAL AND PHARMACEUTICAL STUDENTS, ETC. By FREDERIC J. FARRE, M.D., Cantab., F.L.S., Fellow of the Royal College of Physicians of London, Honorary Member of the Pharmaceutical Society of Great Britain, Examiner in Materia Medica and Pharmacy to the University of London, etc., etc. Assisted by ROBERT BENTLEY, M.R.C.S., F.L.S., Honorary Fellow of Kings College, London, Prof. of Botany, etc., etc.; and by ROBERT WARINGTON, F.R.S., F.C.S., Chemical Operator to the Society of Apothecaries, and Vice-President of the Chemical Society. Edited, with numerous references to the U.S. Pharmacopœia, and many other additions, by HORATIO C. WOOD, Jr., M.D., Prof. of Botany, University of Pennsylvania, Auxiliary Faculty of Medicine; Member of the American Phil. Society, etc., etc., etc., etc. With 236 wood engravings. Philadelphia: HENRY C. LEA. 1866.

This, though an abridgement of Dr. PEREIRA'S original work, is, nevertheless, a ponderous volume of 1030 pages. It is published in good style, in substantial leather binding. The four editors and assistant editors, British and American, have so altered, abridged, and modified the original work, that we doubt whether, if Dr. PEREIRA were alive, he would recognize much in the present edition as his own work. It is, however, a convenient and very valuable treatise on materia medica.

For sale by W. B. KEEN & Co., 148 Lake Street.

